# Clinical instability of breast cancer markers is reflected in long-term *in vitro* estrogen deprivation studies

**DOI:** 10.1186/1471-2407-13-473

**Published:** 2013-10-11

**Authors:** Jelena Milosevic, Johanna Klinge, Anna-Lena Borg, Theodoros Foukakis, Jonas Bergh, Nicholas P Tobin

**Affiliations:** 1 Cancer Center Karolinska, Karolinska Institutet and University Hospital, Stockholm S-171 76, Sweden; 2 Department of Oncology and Pathology, Radiumhemmet, Karolinska Institutet and University Hospital, Stockholm, Sweden; 3 Honorary Professor, Manchester University, Manchester M20 4BX, England

## Abstract

**Background:**

Long-term estrogen deprivation models are widely employed in an *in vitro* setting to recapitulate the hormonal milieu of breast cancer patients treated with endocrine therapy. Despite the wealth information we have garnered from these models thus far, a comprehensive time-course analysis of the estrogen (ER), progesterone (PR), and human epidermal growth factor 2 (HER-2/neu) receptors on the gene and protein level, coupled with expression array data is currently lacking. We aimed to address this knowledge gap in order to enhance our understanding of endocrine therapy resistance in breast cancer patients.

**Methods:**

ER positive MCF7 and BT474 breast cancer cells were grown in estrogen depleted medium for 10 months with the ER negative MDA-MB-231 cell line employed as control. ER, PR and HER-2/neu expression were analysed at defined short and long-term time points by immunocytochemistry (ICC), and quantitative real-time RT-PCR (qRT-PCR). Microarray analysis was performed on representative samples.

**Results:**

MCF7 cells cultured in estrogen depleted medium displayed decreasing expression of ER up to 8 weeks, which was then re-expressed at 10 months. PR was also down-regulated at early time points and remained so for the duration of the study. BT474 cells generally displayed no changes in ER during the first 8 weeks of deprivation, however its expression was significantly decreased at 10 months. PR expression was also down-regulated early in BT474 samples and was absent at later time points. Finally, microarray data revealed that genes and cell processes down-regulated in both cell lines at 6 weeks overlapped with those down-regulated in aromatase inhibitor treated breast cancer patients.

**Conclusions:**

Our data demonstrate that expression of ER, PR, and cell metabolic/proliferative processes are unstable in response to long-term estrogen deprivation in breast cancer cell lines. These results mirror recent clinical findings and again emphasize the utility of LTED models in translational research.

## Background

The pathogenesis of breast cancer is a complex, multistep process involving multiple genetic changes. A major risk factor associated with the development of the disease is the duration of exposure to estrogens, the length of which is increased in women experiencing early menarche and/or late menopause. Estrogens are steroid hormones that play important roles in the growth and development of the mammary gland and it is well established that the growth of breast cancer cell lines in culture or in ovariectomized nude mice is stimulated by estrogens [1-3].

Approximately two-thirds of all breast cancer tumours are ER-positive [4-6] and more than 50% of these are also PR-positive [7]. Both receptors are useful in predicting response to endocrine therapy [5,7-9] and in general ER-negative tumours are associated with early recurrence and poor patient survival relative to those that are ER-positive [5,8,9]. Despite clinical advances of ER-targeted therapy, *de novo* and acquired resistance to all forms of endocrine therapy remains a great obstacle [8,9]. Complicating matters, we and others have shown in mostly retrospective studies, that expression of ER and PR are unstable during tumour progression from a primary lesion to its corresponding metastasis [10-13].

Long-term estrogen deprived (LTED) cell lines can serve as an *in vitro* model mimicking the hormonal milieu of breast cancer cells in oophorectomized pre-menopausal women, postmenopausal women and/or patients treated with primary endocrine therapy, in particular aromatase inhibitors (AIs) [14]. Of note, the use of AIs in place of traditional endocrine treatments results in a statistically significant survival gain (HR 0.90, 95% CI 0.84 to 0.97) [15].

Whilst previous studies have examined ER, PR and HER-2/neu expression in an LTED setting, no comprehensive gene and protein analysis has been performed on all three markers. As such, our descriptive study addresses this knowledge gap by determining the levels of ER, PR and HER-2/neu gene and protein expression in two ER-positive and one ER-negative cell line at multiple time points, coupled with gene expression array profiling, all in a well-described LTED model [16-20]. Adding further clinical relevance to our analysis, we related our expression array findings to publicly available array data of breast cancer patients treated with an aromatase inhibitor. Our work highlights the unstable nature of ER and PR expression under conditions of estrogen deprivation, and demonstrates the significant overlap of genes altered in LTED cell lines and AI-treated patients.

## Methods

### Cell culture

A long-term estrogen deprivation (LTED) model was used to study the three commonly used breast cancer cell lines MCF7, BT474 and MDA-MB-231 [7,8]. MCF7 and MDA-MB-231 cells were newly purchased from Sigma-Aldrich and BT474 cells from the American Type Culture Collection (ATCC). Control and LTED cells were routinely maintained in phenol red containing MEM or DMEM supplemented with 10% fetal bovine serum (FBS) or phenol red-free MEM or DMEM supplemented with 10% dextran-coated charcoal-stripped FBS (DCC-FBS) to remove substantial amounts of estrogen, respectively. Each culture medium was further supplemented with 100 IE/ml penicillin and 100 μl/ml streptomycin. All cells were grown at 37°C in a humidified atmosphere of 5% CO_2_ and 95% air.

### Immunocytochemistry

50 000 cells per cell line (MCF7, BT474 and MDA-MB-231 cells) were attached to slides (ChemMateTM Capillary Gap Microscope Slides, DAKO) by centrifuging them in a Cytospin 3 centrifuge (Shandon, Thermo Electron corporation, Waltham, Massachusetts), at 1000 rpm for 4 minutes in room temperature. The slides were then fixed in 4% formalin for 10 minutes at room temperature, followed by PBS for 10 minutes, methanol for 4 minutes in -20°C, and acetone for 1 minute in -20°C, before being placed in TBS. Automatic immunostaining was performed in a DAKO Tech Mate instrument (DAKO, Glostrup, Denmark). Staining of ER and PR was done using the recommended DAKO ChemMate Detection Kit (Peroxidase/DAB Rabbit/Mouse). The MDA-MB-231 cell line served as negative control for ER, PR and HER-2/neu expression. MCF7 cell line was used as positive control for ER expression, while BT474 cell line served as positive control for PR and HER-2/neu expression.

Immunoslides were assessed in a microscope by counting of positive cells and degree of staining. We used a modified H score system, using the formula: H score = (0 ×% tumour cells negative) + (1.5 ×% tumour cells moderately positive) + (3 ×% tumour cells strongly positive), giving a range 0–300. Five hundred cells were counted per slide. Two observers (JM and JK) evaluated the immunoslides, and the final score was calculated by taking the mean score. If the ratio between two scores was higher than 1.5, the slides were re-evaluated to reach consensus.

The following primary antibodies were used for immunocytochemical analyses: Monoclonal mouse anti-human progesterone receptor (PR) antibody (Clone PgR 636, DAKO, Glostrup, Denmark), diluted 1:1000, monoclonal mouse anti-human estrogen receptor antibody NCL-ER-6 F11 (NovoCastra Laboratories Ltd, Newcastle, UK), diluted 1:50, monoclonal mouse anti-human HER-2 (c-erbB-2 Oncoprotein) antibody (NCL-CB11), diluted 1:250 (Novocastra Laboratories Ltd., Newcastle, UK).

### RNA isolation

RNA extraction was performed according to the RNeasy mini protocol (Qiagen, Germany). Briefly, one to five millions cells were collected for isolation of RNA from each sample before being applied to the MicroSpin affinity columns in the Qiagen kit. The quality of RNA was assessed using an Agilent 2100 bioanalyzer (Agilent Technologies, Rockville, MD, USA).

### Quantitative real-time PCR analysis

The mRNA expression levels of *ESR1*, *PGR*, *ERBB2* and an endogenous housekeeping gene encoding for 18S ribosomal RNA as a reference were quantified using TaqMan® technology on an ABI PRISM 7500 sequence detection systems (PE Applied Biosystems). Sequence-specific primers and probes were selected from the Assay-on-Demand products (Applied Biosystems), including *ESR1* (assay ID: Hs01046817_m1), *PGR* (Hs00172183_m1), *ERBB2* (Hs00170433_m1) and *18S* ribosomal RNA (Hs99999901_s1). All qRT-PCR experiments included a no template control and were performed at least in duplicate.

### Microarray analysis

A total of eight samples, four from each cell line (control, 2 days, 6 weeks and 10 months), were selected for microarray analysis, performed at the core facility for Bioinformatics and Expression Analysis (BEA) at Karolinska Institutet. Briefly, biotinylated cRNA was hybridized to HG-U133 Plus 2.0 oligonucleotide arrays (Affymetrix Incorporated, Santa Clara, CA, USA), washed and scanned according to the protocol recommended by the supplier. Gene Chip Operating Software (GCOS) was used for calculation of detection calls, signal values and for calculation of the target intensity scaling of each array to an identical value and quantification of the signal log ratio. An average signal log ratio value was calculated for all transcripts in the long-term estrogen deprived cell lines compared to the cell lines cultured in medium containing estrogen. A minimum signal log ratio of 0.7 in each of four pair-wise comparisons was set as a threshold for significant differential expression. The quality of the data was verified by correlation analysis and multidimensional scaling plots in *R* statistical environment using Bioconductor packages ( http://www.bioconductor.org). This data has been made publically available at NCBI GEO with series accession number GSE50820. Gene Ontology (GO) terms enriched in the lists of up-regulated and down-regulated genes including the 300 genes with highest SLR, were identified by Fisher’s exact test. For comparison of genes significantly changed in response to estrogen silencing to those significantly altered in our LTED model, we accessed publically available data (Gene expression omnibus number: GSE27473) from the NCBI GEO repository. The data is taken from a publication by Al Saleh *et al*. [21] where gene expression changes are determined in MCF7 cells after estrogen receptor silencing. In order to directly compare with our data, we downloaded and re-analysed the dataset using the statistical parameters outlined above to determine genes significantly changed in response to estrogen silencing.

### Statistical analysis

All statistical analysis were performed using SPSS data analysis statistics software system version 17.0 (SPSS Inc., Chicago, IL, USA), the statistics tool in Microsoft Excel or R. ANOVA with post-hoc Tukey was performed on H-score and qPCR data and significance was calculated relative to day 0 control. Experimental results are expressed as mean ± SEM, where applicable. *P*-values of < 0.05 were considered statistically significant.

## Results

### Re-expression of ER in an estrogen deprived environment occurs in the absence of PR in MCF7 cells

**Figure 1:**
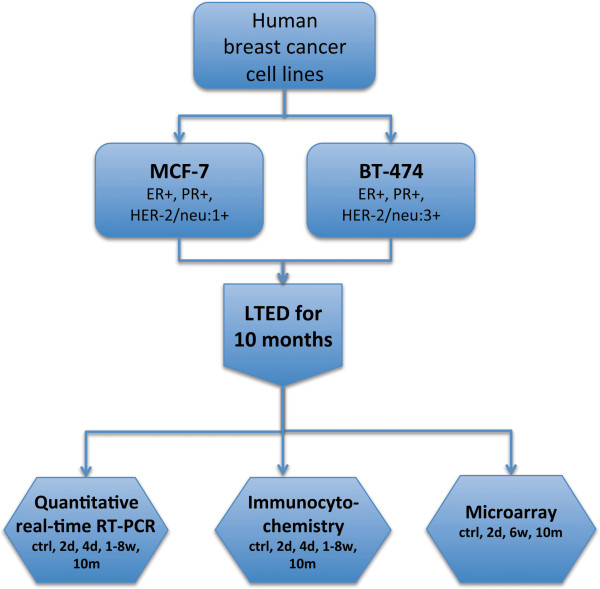
**Experimental flowchart.** ER, PR and HER-2/neu expression were analysed by qRT-PCR and immunocytochemistry (ICC) at selected time points (control, 2 days, 4 days, 1–8 weeks and 10 months after estrogen deprivation). Microarray analysis was performed on control cells and at 2 days, 6 weeks and 10 months after estrogen deprivation.

The breast cancer cell lines MCF7 and BT474 were cultured without estrogen for up to 10 months and examined by immunocytochemistry (quantified by H-score) and qRT-PCR for changes in expression of ER, PR and HER-2/neu at the time points shown in our experimental overview (Figure 1). The ER, PR and HER-2/neu negative MDA-MB-231 cell line served as negative control. Cultured without estrogens, both ER positive cell lines initially stopped growing but MCF7 cells had returned to control levels of growth after ten months of continuous culture as determined by Ki67 (Additional file 1: Figure S1, upper panel), in line with previous studies [22]. BT474 cells displayed increased Ki67 expression after 10 months in LTED culture relative to 6 weeks, but had still not returned control levels of proliferation (Additional file 1: Figure S1, lower panel).

**Figure 2:**
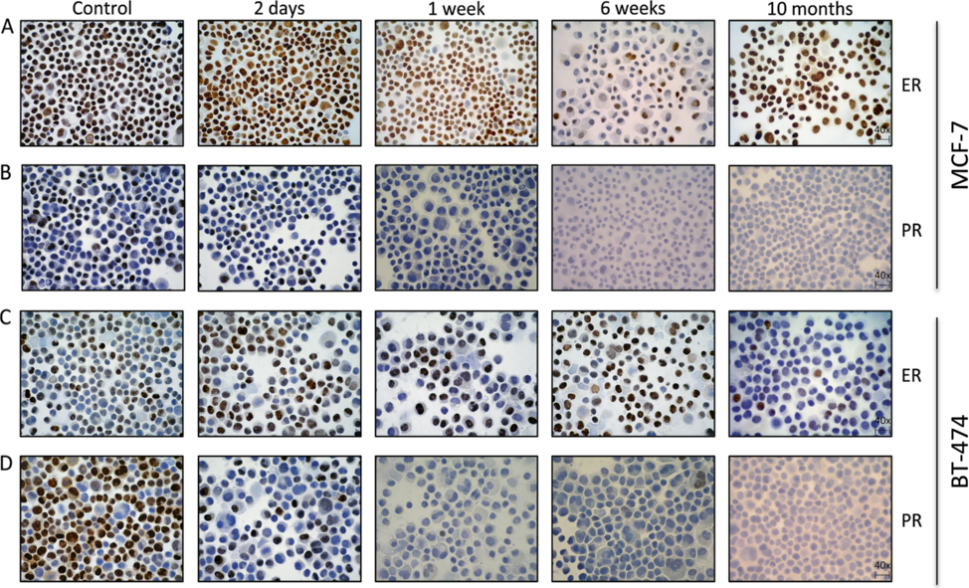
**Characterization of ER and PR expression by ICC at selected time points*. *(A)** ER expression in MCF7 cells. Cells stained brown are positive for the receptor. **(B)** PR expression in MCF7 cells **(C)** ER expression in BT474 cells **(D)** PR expression in BT474 cells. Original magnification 40×.

**Figure 3:**
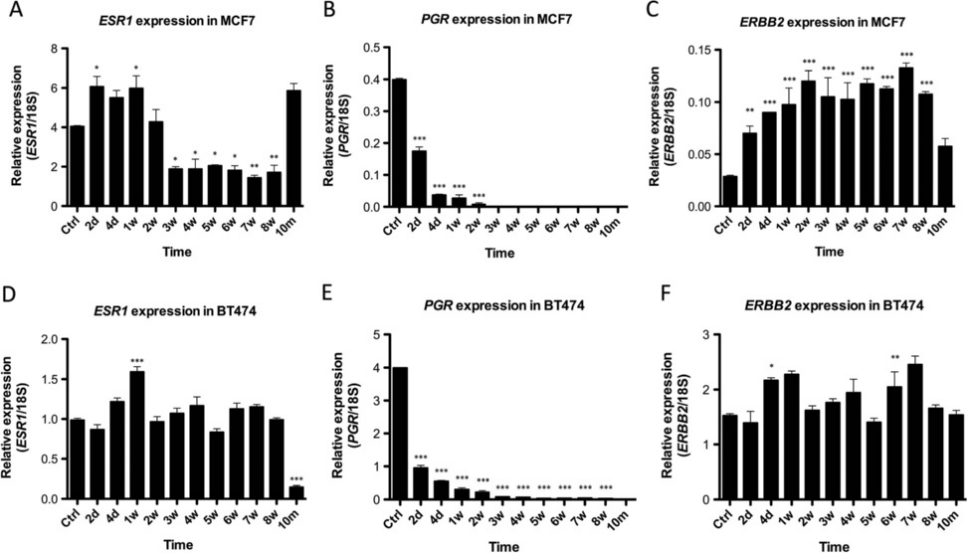
**ESR1, PGR and ERBB2 expression analysed by qRT-PCR. A)** Expression levels of *ESR1*, **(B)***PGR* and **(C)***ERBB2*, respectively, relative to ribosomal 18S in MCF7 cells. **(D)** Expression levels of *ESR1*, **(E)***PGR* and **(F)***ERBB2* relative to ribosomal 18S in BT474 cells. ***P ≥ 0.001, **P ≥ 0.01, *P ≥ 0.05 vs. control, ANOVA with post-hoc Tukey.

ER expression in MCF7 cells decreased gradually from 2 weeks to 8 weeks after estrogen deprivation, but was re-expressed at 10 months as determined by immunocytochemistry, qRT-PCR and H-score (Figures 2A, 3A and Additional file 2: Figure S2A, respectively). Using identical methods, we found PR significantly down-regulated 2 days after estrogen deprivation (Figures 2B, 3B and Additional file 2: Figure S2B). After 1–2 weeks its expression was no longer detectable and remained so for the 10 month duration of the study. Changes in ER and PR protein expression at early time points were also confirmed by western blot (Additional file 3: Figure S3). Whilst we noted no change in HER-2/neu expression in response to estrogen deprivation by ICC (Additional file 4: Figure S4- upper panel), we did find a small increase at the mRNA level (Figure 3C). It should be highlighted however, that given the scale of *ERBB2* expression it is unsurprising that this increase is not reflected by ICC. No expression of ER, PR or HER-2/neu was found in MDA-MB 231 cells as determined immunocytochemically (Additional file 5: Figure S5).

These results demonstrate the ability of MCF7 cells to up-regulate ER in the absence of PR under LTED conditions and moreover, highlight the unstable nature of ER and PR expression on both the gene and protein level when external estrogen levels are depleted.

### Reduced ER and PR expression in BT474 cells exposed to long-term estrogen deprived conditions

In BT474 cells, no clear trend in ER expression was noted during the first 8 weeks of estrogen deprivation, but a significant reduction was noted after 10 months by ICC and qRT-PCR (Figures 2C, 3D and Additional file 2: Figure S2C). Similarly to MCF7 cells, PR expression fell dramatically following 2 days of estrogen deprivation, was no longer expressed after 2–3 weeks, and remained undetectable on the protein level for the remainder of the study (Figures 2D, 3E and Additional file 2: Figure S2D). These changes were also confirmed by western blot at early time points (Additional file 3: Figure S3). HER-2/neu expression was not changed at any of the tested time points by ICC (Additional file 4: Figure S4, lower panels) but a weak trend towards increased *ERBB2* was seen in the first 8 weeks of estrogen deprivation by qRT-PCR (Figure 3F).

These experiments again emphasize the instability of ER and PR in response to estrogen deprivation. Moreover, whilst the reduction in ER after 10 months in BT474 cells contrasts its increased expression at the same time-point in MCF7 cells, these results are consistent with the idea that individual cell lines can respond differently to LTED conditions [23].

### Metabolic and cell cycle related genes down-regulated in initial response to estrogen deprivation are re-upregulated in long-term culture

Gene expression profiles were analysed in MCF7 and BT474 cells at 0 and 2 days, 6 weeks and 10 months after estrogen deprivation in order to examine gene expression changes in response to estrogen deprivation (see Figure 1, experimental overview).

**Figure 4:**
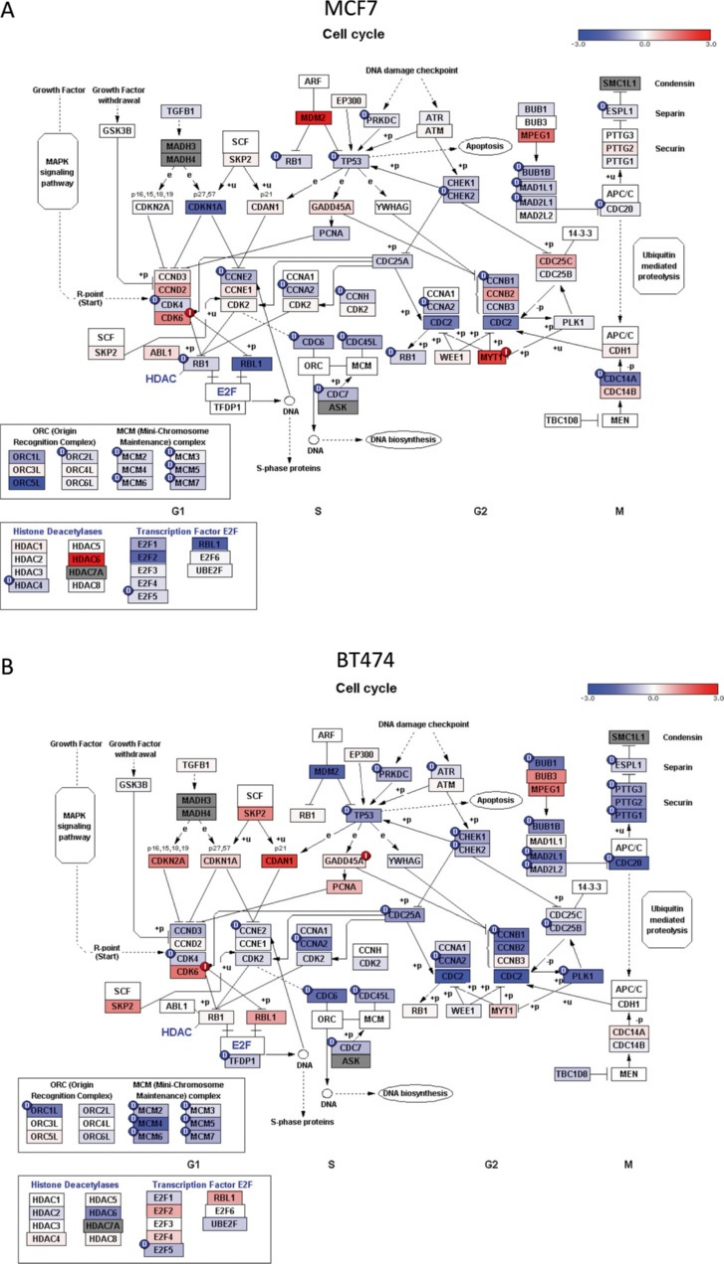
**Cell cycle genes affected in estrogen deprived cells.** Red and blue boxes indicate up- and down regulated gene expression in response to estrogen deprivation relative to untreated control. Small circles (red and blue) mark a statistically significant increase or decrease in expression. **(A)** MCF7 cells at 2 days after estrogen deprivation versus control cells. **(B)** BT474 cells at 6 weeks after estrogen deprivation versus control cells.

In MCF7 cells, when comparing the 2 day and 6 week time points to control (0 days), the most down-regulated genes were those involved in metabolic processes and cell cycle, as expected. Figure 4A depicts the genes affected in cell cycle after 2 days and similar results were noted after 6 weeks (Additional file 6: Figure S6A). A full list of the cell cycle gene altered in MCF7 cells after 2 days LTED is provided in Additional file 7: Table S1. In the same samples the most notable up-regulated genes were *TIMP2*- which is involved in negative regulation of cell proliferation and *NOTCH1*. In the 10 month vs. control comparison we noted a reversal of these trends and genes involved metabolic and proliferative processes were up-regulated. Interestingly, genes down-regulated in the 10 month samples included those putatively involved in cell migration and motility (*DNALI1, DNAH1, CXorf61*), the apoptotic gene *SULF1* and the PR gene *PGR*. Of note, a similar study of gene changes in MCF7 LTED cells over time has been previously performed, albeit in shorter time frame of 180 days [24]. Despite this difference, we saw a similar effect of LTED on the expression of the *ESR1*, *MKI67*, *EGFR* and *RAF1* genes in our study as that found in the work of Aguilar *et al*. (Additional file 8: Figure S7), highlighting the reproducibility of LTED models.

In general, similar results were noted for BT474 cells at early time points, however the effect on cell cycle was less obvious after 2 days (Additional file 7: Figure S6B) but became apparent after 6 weeks (Figure 4B). A full list of the cell cycle gene altered in BT474 cells after 6 weeks LTED is provided in Additional file 9: Table S2. Again, the 10 month vs. control comparison showed up-regulation of genes involved in metabolic and proliferative processes (*CXCR4, INHBA*), and down-regulation of those involved in cell motility (*KIF6, VIM, TUBA1A*), apoptosis (*SEMA6A*) and the *PGR* gene.

Together, these results point to an early down-regulation of genes involved in metabolic processes and cell cycle, as would be expected from estrogen deprivation. In long-term LTED culture, the situation is reversed and genes involved in the same processes are up-regulated whilst notably, genes implicated in cell motility and epithelial-to-mesenchymal transition (*VIM*) are down-regulated, in line with the “go or grow” hypothesis [25].

### Strong similarity between cell line genes altered in response to estrogen deprivation and those found in AI-treated breast cancer patients

Next, with the aim of comparing the observed gene expression changes following estrogen deprivation in breast cancer cells to patients who received aromatase inhibitor (AI) treatment, we analysed a publicly available array data set consisting of 58 postmenopausal breast cancer patients with array profiles assessed before and after neoadjuvant treatment with letrozole (Gene expression omnibus number: GSE5462) [26].

In order to determine if similar processes were affected between our cell lines in response to estrogen deprivation and AI treated patients, we performed gene ontology analysis on our day 2 vs. control gene expression from MCF7 (Additional file 10: Table S3) and BT474 (Additional file 11: Table S4) cells. We found that the most changed processes in our cell line model including metabolic pathways, cell cycle, DNA replication, developmental processes and ion transport were also significantly changed in AI treated patients (see Miller *et al.*[26]).

**Figure 5:**
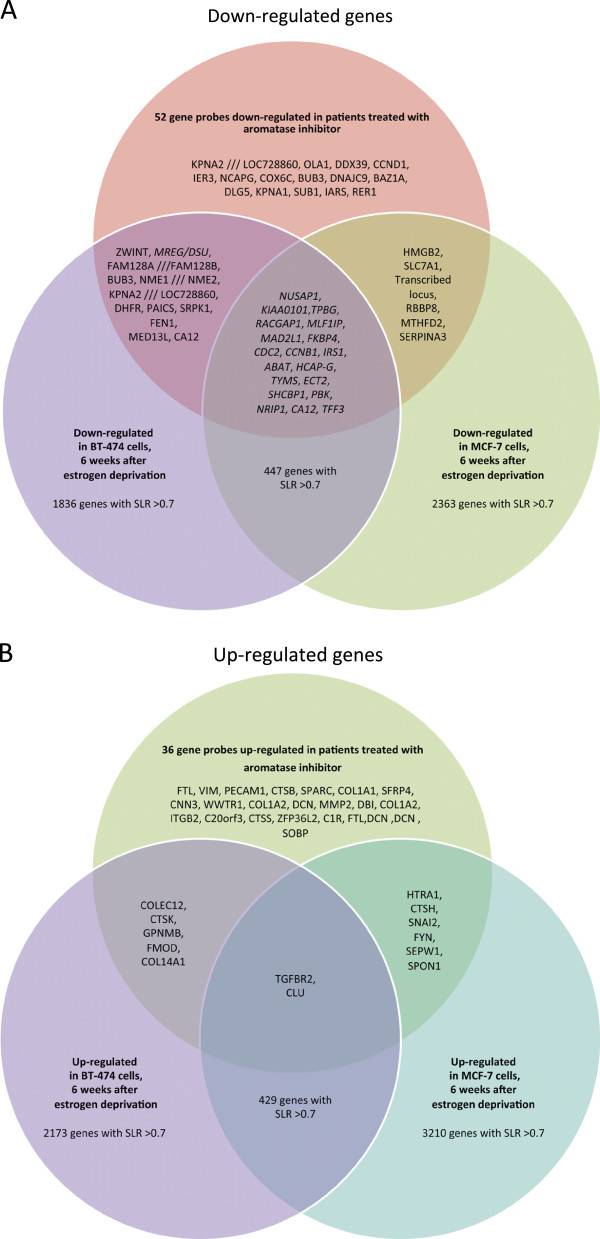
**Venn diagram comparing significantly changed genes in MCF7, BT474 cell lines and AI-treated patients. (A)** Genes significantly down-regulated in response to estrogen deprivation after 6 weeks vs. control in MCF7 and BT474 cells compared with those significantly down-regulated in AI-treated patients **(B)** Genes significantly up-regulated in response to estrogen deprivation after 6 weeks vs. control in MCF7 and BT474 cells compared with those significantly up-regulated in AI-treated patients.

Next, we examined the specific genes that were differentially expressed in our cell line model with those significantly changed upon letrozole treatment (Miller *et al.*[26]). We found that 14 of the 52 genes displaying decreased expression in AI-treated patients were also down-regulated in MCF7 cells after 2 days. This number rose to 25 out of 52 when considering genes down-regulated in MCF7s 6 weeks after estrogen deprivation (Figure 5A).

Similarly in BT474 cells after 2 days, only 2/52 genes overlapped with those down-regulated in AI patients, but this increased to 31/52 when comparing to the 6 week estrogen deprived (Figure 5A) samples. Of note, 19/52 gene probes down-regulated in both BT474 and MCF7 cells at 6 weeks after estrogen deprivation were also down-regulated in AI-treated patients (Figure 5A).

Up-regulated genes showed a smaller overlap with patient data; in MCF7 cells 4/36 and 8/36 gene probes up-regulated after 2 days and 6 weeks estrogen deprivation respectively were also up-regulated in AI treated patients (Figure 5B). In BT474 cells these numbers fell to 2/36 and 7/36 gene probes after 2 days and 6 weeks respectively (Figure 5B). Two genes were up-regulated in both MCF7 and BT474 cells at 6 weeks (*TGFBR2* and *CLU*) were also upregulated in AI treated patients (Figure 5B).

Finally, in order to determine if gene changes caused specifically by loss of estrogen receptor are also present in the genes of LTED cells and AI –treated patients, we utilised publically available data (Gene expression omnibus number: GSE27473) of MCF7 cells treated with siRNA against the estrogen receptor [21]. Notably, we found an overlap of 4 genes significantly up-regulated and 11 genes significantly down-regulated in all three datasets (Additional file 12: Table S5). Of the up regulated genes, both *SNAI2* and *TGFBR2* are associated with promotion of epithelial-to-mesenchymal transition, whilst among the down-regulated genes were those responsible for the suppression of EMT including *RACGAP1*, *TFF3* and *IRS1*. These results again implicate the induction of EMT through loss of estrogen receptor, in line with the work of others [21].

Taken together these data lend weight to the ability of this established model to provide relevant translational information and further support its use as a testing ground for elucidation of factors that mediate anti-estrogen treatment resistance.

## Discussion

In spite of the substantial progress that has been achieved in recent years in the treatment of hormone receptor positive breast cancer, *de novo* and acquired resistance to endocrine therapy is still a major clinical problem [8,9]. In this descriptive study, we employed a LTED model to gain a greater understanding of how estrogen deprivation impacts clinically relevant prognostic markers and gene expression over time. To our knowledge, this is the first report to comprehensively investigate ER, PR and HER-2/neu expression along with qRT-PCR and gene expression array profiles at multiple early and late time points, in breast cancer cell lines after estrogen deprivation. Overall, our data are in line with previous reports showing that breast cancer cells can survive estrogen deprivation and re-grow, creating a phenotype that is likely less responsive to anti-hormonal therapy [27]. Additionally, due to the multiple consecutive time points examined, we note clear trends in how the expression of ER and PR change over time on both the gene and protein level. Lastly, we underline the similarities between the specific genes changed in our LTED cell lines and patients treated with aromatase inhibitors, demonstrating the strong translational value of this model, as others have also noted [23,24,28].

In order to put our work in the context of other studies and strengthen our findings, we compared our gene expression results to that of Aguilar *et al*., who performed a similar study in an MCF7 LTED model [24]. Through integrated aCGH and gene expression analysis the Aguilar study demonstrated that there may be shift towards a transcriptomic program in LTED cells that is independent of ERα transcriptional function. Whilst we did not perform matching aCGH analysis on our LTED samples, and despite the differences in time points assessed in both studies, we did note similar changes in gene expression probes over time. Specifically, we noted analogous changes in the probes for *ESR1, MKI67*, *EGFR* and *RAF1* (but not *GATA3*), thus lending support to hypotheses proposed by Aguilar *et al.*

Recent publications including two prospective studies, indicate lack of stability of ER and PR during tumour progression, in particular they seem to be altered when adjuvant therapies are given [29-31]. This loss of receptors, at least in the examined parts of the biopsies, may be a further factor involved in resistance to endocrine therapies. It is also apparent from these studies that ER and PR seem to be more discordant in patients receiving more abundant adjuvant therapies and a similar finding has been demonstrated with chemotherapy and trastuzumab in the comparison of HER-2/neu status in the primary tumour and the corresponding recurrence [31]. This clinical instability is reflected in our present cell line model, again underlining the suitability of LTED studies for investigating the time related alteration of receptors during conditions which mimic endocrine therapy with aromatase inhibition.

Previous studies have shown the propensity of breast cancer cells to adapt to conditions of long-term estrogen deprivation by up-regulating expression of ER, but not PR [19,32], thus developing hypersensitivity to the mitogenic effect of estradiol. In our experiments, we observed a marked up-regulation of ER in the MCF7 but not BT474 cell line at 10 months after estrogen deprivation. Some reports claim that this estradiol hypersensitivity is not a consequence of ER-mediated gene transcription but rather related to activation of the MAPK/ERK [19] and EGFR/ERBB/AKT pathways [24]. Similarly, recent evidence has also implicated a switch from ERα to NOTCH signalling in LTED cells [28], a finding supported by our analysis where we see an up-regulation of the *NOTCH1* in MCF7 cells relative to control after 6 weeks of LTED culture.

The up-regulation of *NOTCH1* fits well with our findings of increased expression of genes that promote EMT in both LTED MCF7 cells at 6 weeks and AI treated patients. Previous studies have linked induction of EMT under hypoxic conditions to Notch signalling [33], whilst ectopic expression of Notch1 intracellular domain (N1CD) has been demonstrated to trigger an EMT in epithelial cancer cells [34]. Of particular note, others have shown that a decrease in estrogen dependency is correlated with an increase of the EMT marker Snail1 in an MCF7 LTED model [35]. What these results mean in the context of AI treatment of breast cancer patients is difficult to ascertain. One might expect that as induction of EMT leads to an enhancement in the migratory capacity of cells, treating breast cancer patients with AIs would push tumour cells towards a more invasive metastatic phenotype. However, given the high success rates of endocrine treatments and reduced numbers of metastasis seen amongst these patients (relative to those who receive chemotherapy), this hypothesis would seem unlikely.

The down-regulation of PR following estrogen deprivation observed in our experiments could be caused by multiple cellular mechanisms. Cui *et al.* have shown that insulin-like growth factor-1 (IGF-1), independent of ER activity, considerably down-regulates PR through the PI3K (Akt/mTOR) pathway [36]. Along with others, they propose that low PR status may serve as an indicator of substantial activation of the growth factor signalling cascade, leading to hormonal therapy resistance [37-40]. However, our gene array data did not support any significant involvement of the PI3K/Akt pathway and as such the mechanisms governing loss of PR in our model will require further investigations.

## Conclusions

Our data highlight the instability of ER, PR and metabolic/proliferative processes in response to short and long-term estrogen deprivation. Additionally we demonstrate considerable the overlap between genes altered in LTED culture and AI-treated breast cancer patients. These results further strengthen the use of LTED models as a valuable translational research tool to further our understanding of the major clinical obstacle that is hormonal resistance.

## Competing interests

Professor Bergh receives research funding from Merck, paid to Karolinska Institutet and from Amgen, Roche, Sanofi-Aventis and Bayer, paid to Karolinska University Hospital. The authors have no other potential competing interest to disclose.

## Authors’ contributions

JM carried out the cell culturing, immunocytochemistry, H score analysis and qRT–PCR analysis, performed statistical analysis studies, participated in the interpretation of the data and drafted the manuscript. JK carried out H score analysis, participated in the interpretation of the data and helped to draft the manuscript. ALB helped with the immunocytochemistry analysis and isolation of RNA and DNA. TF participated in the qRT–PCR analysis. JB conceived of the study, participated in its design and coordination and revised the manuscript critically. NT participated in the interpretation of the data, design, performed the statistical analysis, and revised the manuscript critically. All authors read and approved the final manuscript.

## Pre-publication history

The pre-publication history for this paper can be accessed here:

http://www.biomedcentral.com/1471-2407/13/473/prepub

## Supplementary Material

Additional file 1: Figure S1. Characterization of Ki67 expression by ICC in MCF7 and BT474 cell lines at consecutive time points. Cells stained brown are positive for Ki67. Upper panel: MCF7 cells, Lower panel: BT474 cells. Original magnification 40×. Click here for file

Additional file 2: Figure S2 Histograms of modified H score analysis. (A) Expression of ER and (B) PR in MCF7 cells. (C) Expression of ER and (D) PR in BT474 cells. ***P ≥ 0.001, **P ≥ 0.01, *P ≥ 0.05 vs. control, ANOVA with post-hoc Tukey. Click here for file

Additional file 3: Figure S3 Western blots showing changes in ER and PR expression in response to estrogen deprivation in BT474 and MCF7 cells at early time points. β-actin is included as loading control and MDA-MB-231 cells are included as negative control for ER and PR expression. Blots are representative. Click here for file

Additional file 4: Figure S4 Characterization of HER-2/neu expression by ICC in MCF7 and BT474 cell lines at consecutive time points. Cells stained brown are positive for HER-2/neu receptor. Upper panel: MCF7 cells, Lower panel: BT474 cells. Original magnification 40×. Click here for file

Additional file 5: Figure S5 Characterization of ER, PR and HER-2/neu expression by ICC in MDA-MB-231 cell line at consecutive time points. (A) Lack of ER expression (B) Lack of PR expression. (C) Lack of HER-2/neu expression. Original magnification 40×. Click here for file

Additional file 6: Figure S6 Cell cycle genes affected in estrogen deprived cells. Red and blue boxes indicate up- and down regulated gene expression in response to estrogen deprivation. Small circles (red and blue) mark a statistically significant increase or decrease in expression. (A) MCF7 cells at 6 weeks after estrogen deprivation versus control cells (B) BT474 cells at 2 days after estrogen deprivation versus control cells. Click here for file

Additional file 7: Table S1 Log fold change of cell cycle genes in MCF7 cells 2 days after estrogen deprivation versus control. This table displays all genes of the human KEGG annotated cell cycle pathway and their fold change after two days of estrogen deprivation relative to control, sorted according to p-value. Note, multiple affymetrix probes can map to the same gene. *Direction of change: I = Increase, D = Decrease and NC = No statistically significant change. Click here for file

Additional file 8: Figure S7 The effect of LTED on selected probesets. Here, in order to put our results in context with other scientific publications we reproduced the probeset plots of Aguilar *et al.* (A) *ESR1* affymetrix probesets (B) *MKI67* affymetrix probesets (C) Genes related to ER genomic function. Click here for file

Additional file 9: Table S2 Log fold change of cell cycle genes in BT474 cells 6 weeks after estrogen deprivation versus control. This table displays all genes of the human KEGG annotated cell cycle pathway and their fold change after 6 weeks of estrogen deprivation relative to control, sorted according to p-value. Note, multiple affymetrix probes can map to the same gene. *Direction of change: I = Increase, D = Decrease and NC = No statistically significant change. Click here for file

Additional file 10: Table S3 MCF7 cells 2 days after estrogen deprivation versus control cells. The table represents the number of genes matching the 10 most commonly occurring GO terms in the GO molecular function, biological processes, and cellular component classes. The 300 genes with highest SLR were selected. Click here for file

Additional file 11: Table S4 BT474 cells 2 days after estrogen deprivation versus control cells. The table represents the number of genes matching the 10 most commonly occurring GO terms in the GO molecular function, biological processes, and cellular component classes. The 300 genes with highest SLR were selected. Click here for file

Additional file 12: Table S5 Genes in common amongst those significantly altered in all three analysed datasets: MCF7 LTED culture, ER-silenced MCF7 cells and breast cancer patients treated with aromatase inhibitors. We determined the genes most significantly altered in three datasets; our MCF7 LTED samples (control vs. 6 weeks, pvalue cutoff = 0.004), a publically available dataset of MCF7 cells where the ER has been silenced (control vs. silenced, pvalue cutoff = 0.004, GSE27473) and a publically available dataset of breast cancer patients treated with aromatase inhibitors (GSE5462). We then determined the genes in common amongst those significantly altered in all three studies and present them here divided into those up and down regulated. Click here for file
