# Supplementary figures and images for: Clinical instability of breast cancer markers is reflected in long-term in vitro estrogen deprivation studies
Source: BMC Cancer. 2013 Oct 11;13:473. doi: 10.1186/1471-2407-13-473 (PMC3852062; doi:10.1186/1471-2407-13-473)

Control

2 days

6 weeks

10 months

MCF-7

40x

Ki-67

BT-474

40x

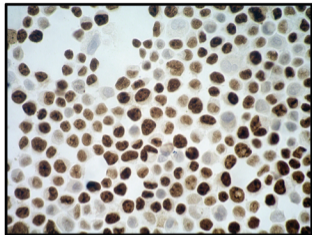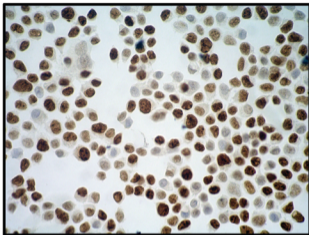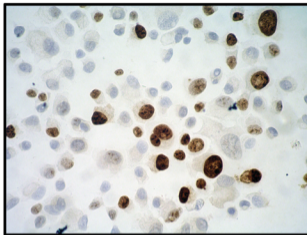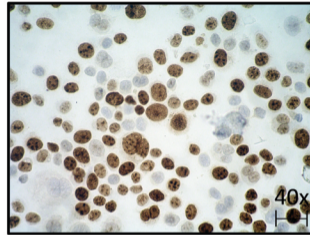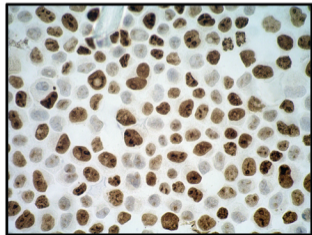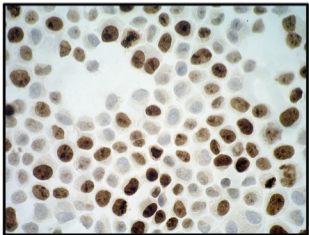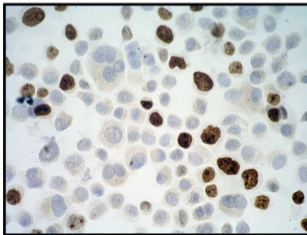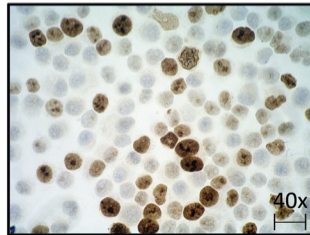

Supplement: Additional file 1: Figure S1. — Characterization of Ki67 expression by ICC in MCF7 and BT474 cell lines at consecutive time points. Cells stained brown are positive for Ki67. Upper panel: MCF7 cells, Lower panel: BT474 cells. Original magnification 40×. [file 1471-2407-13-473-S1.pdf]

A

ER expression in MCF7

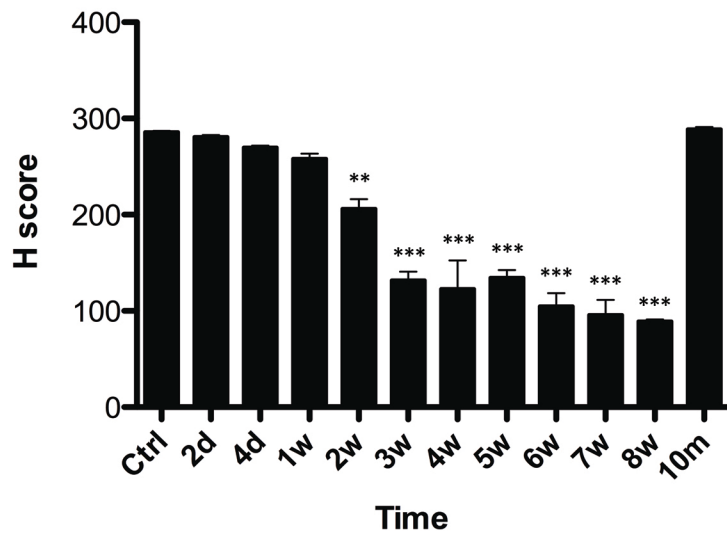

B

PR expression in MCF7

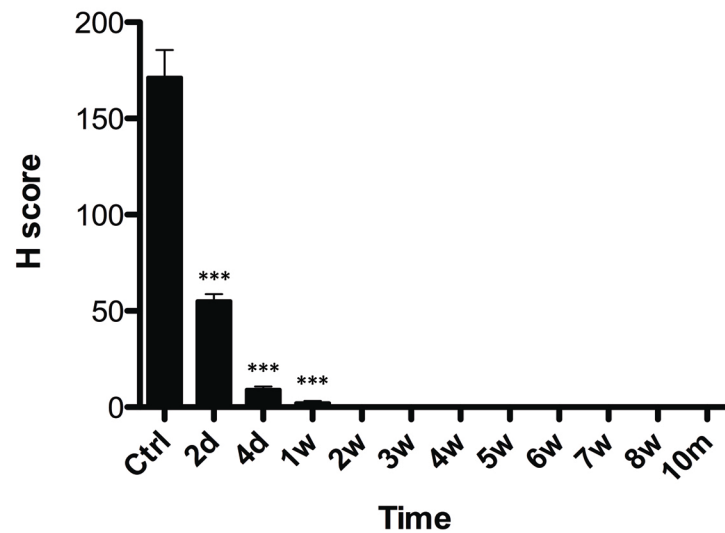

C

ER expression in BT474

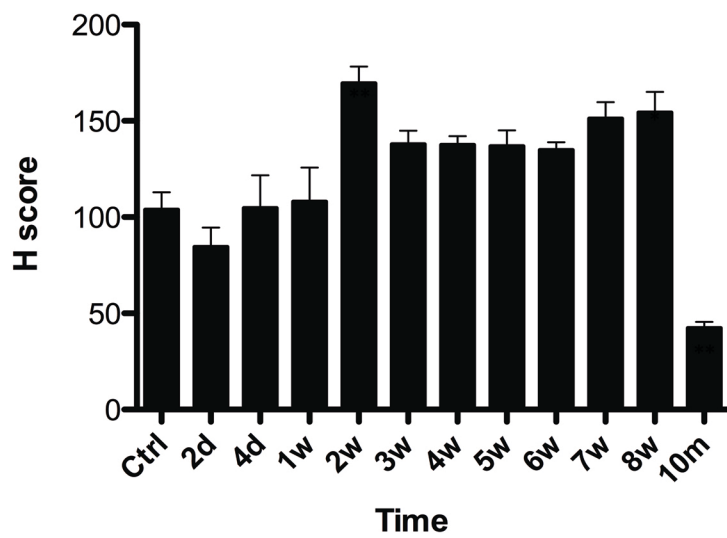

D

PR expression in BT474

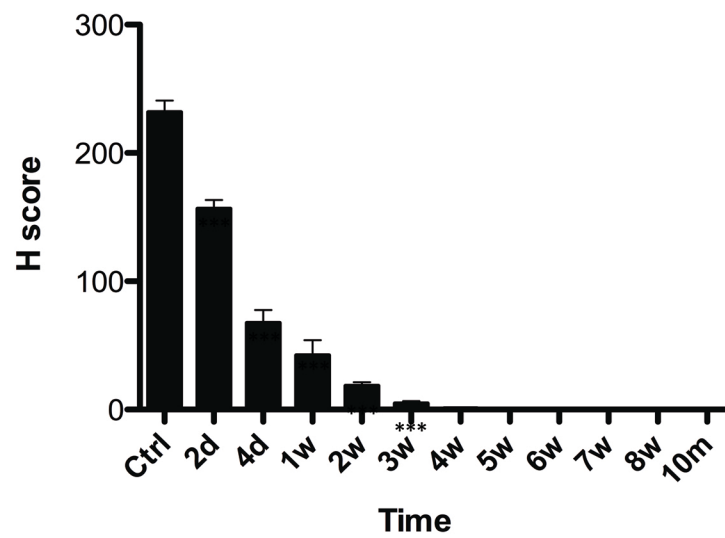

Supplement: Additional file 2: Figure S2 — Histograms of modified H score analysis. (A) Expression of ER and (B) PR in MCF7 cells. (C) Expression of ER and (D) PR in BT474 cells. ***P ≥ 0.001, **P ≥ 0.01, *P ≥ 0.05 vs. control, ANOVA with post-hoc Tukey. [file 1471-2407-13-473-S2.pdf]

# BT474

# MCF-7

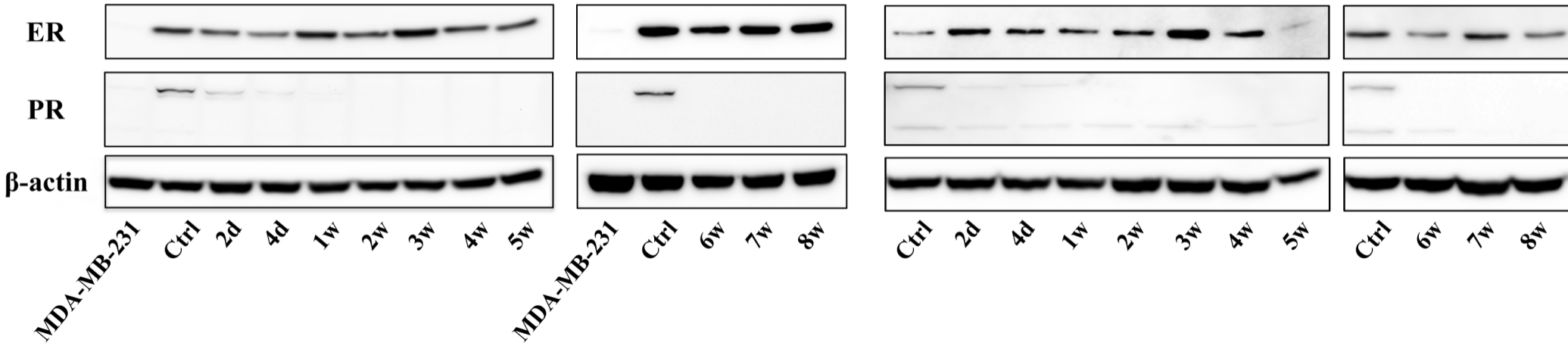

Supplement: Additional file 3: Figure S3 — Western blots showing changes in ER and PR expression in response to estrogen deprivation in BT474 and MCF7 cells at early time points. β-actin is included as loading control and MDA-MB-231 cells are included as negative control for ER and PR expression. Blots are representative. [file 1471-2407-13-473-S3.pdf]

Control

2 days

1 week

6 weeks

10 months

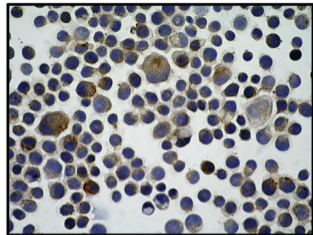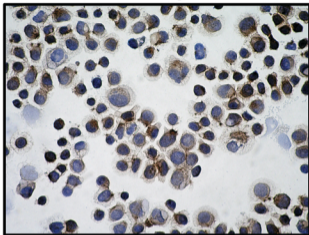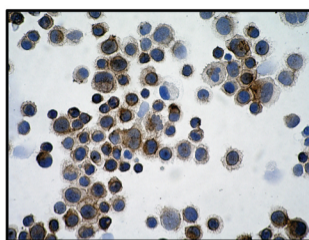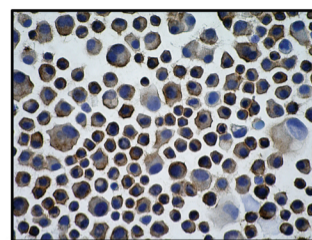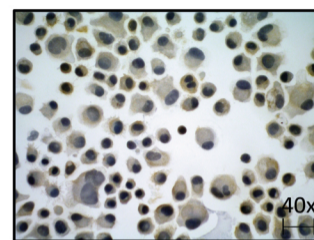

MCF-7

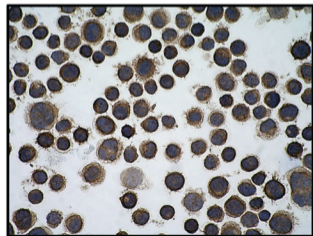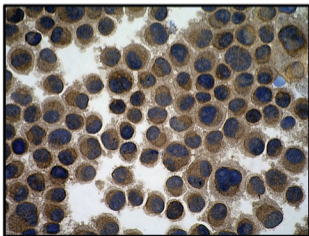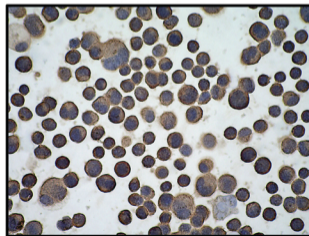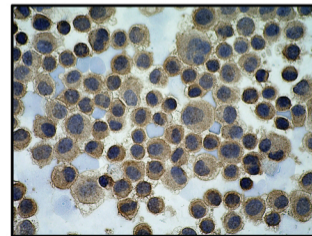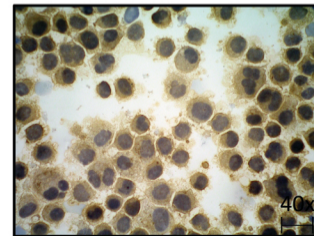

BT-474

HER-/neu2

Supplement: Additional file 4: Figure S4 — Characterization of HER-2/neu expression by ICC in MCF7 and BT474 cell lines at consecutive time points. Cells stained brown are positive for HER-2/neu receptor. Upper panel: MCF7 cells, Lower panel: BT474 cells. Original magnification 40×. [file 1471-2407-13-473-S4.pdf]

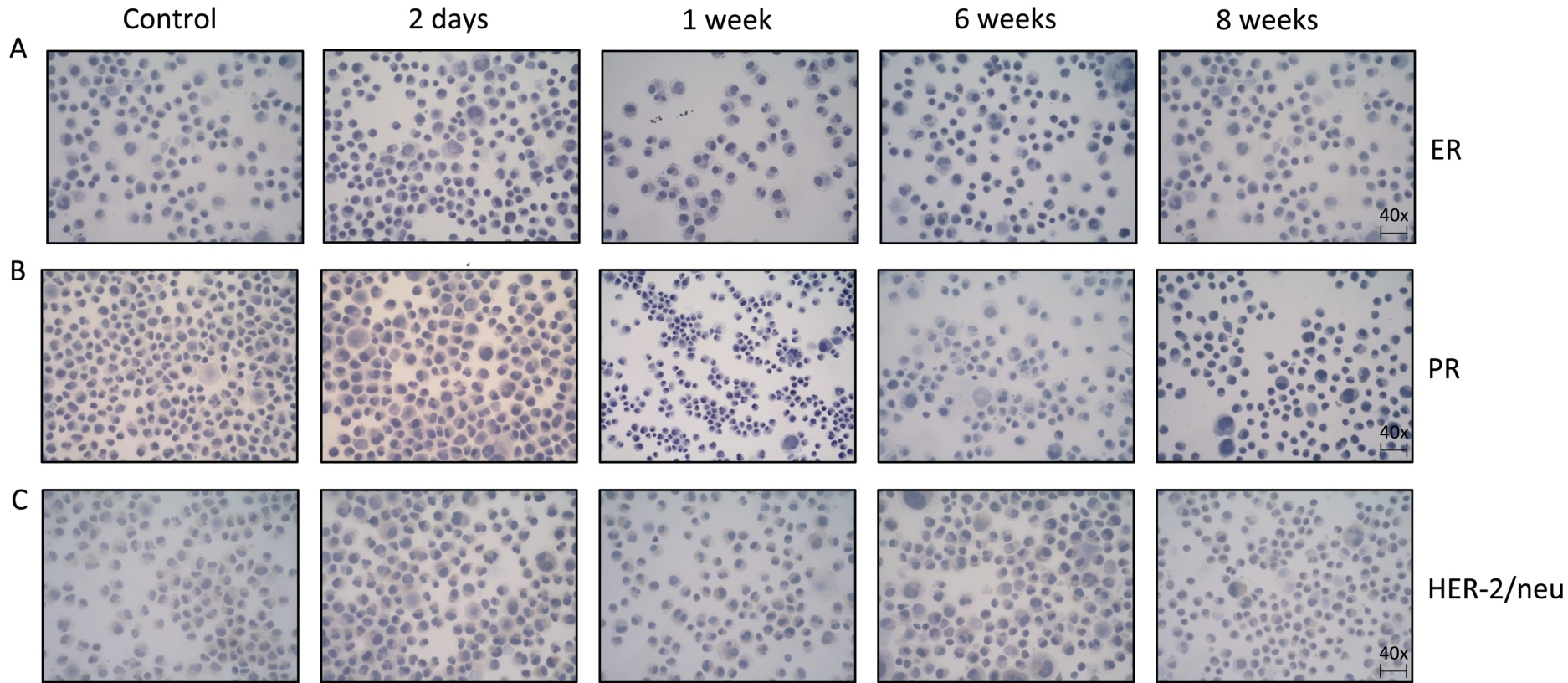

Supplement: Additional file 5: Figure S5 — Characterization of ER, PR and HER-2/neu expression by ICC in MDA-MB-231 cell line at consecutive time points. (A) Lack of ER expression (B) Lack of PR expression. (C) Lack of HER-2/neu expression. Original magnification 40×. [file 1471-2407-13-473-S5.pdf]

A

MCF7

Cell cycle

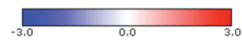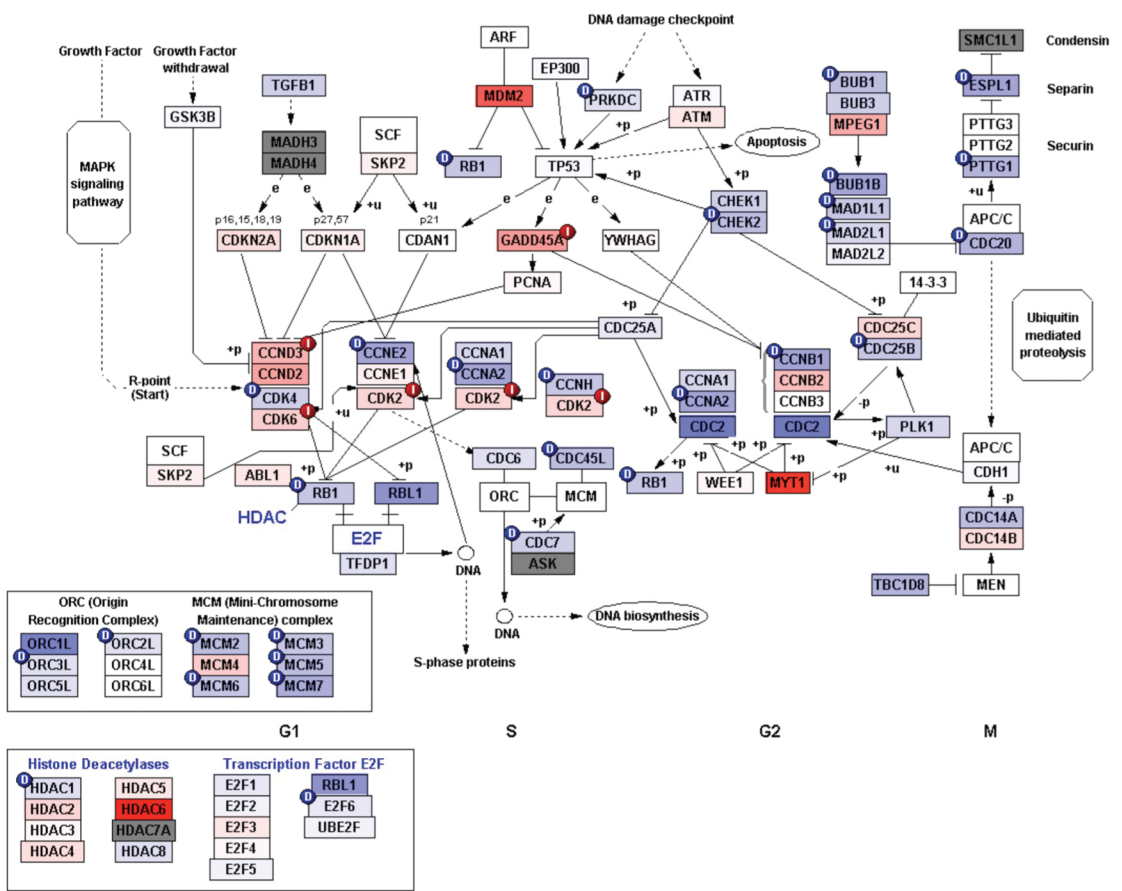

B

BT474

Cell cycle

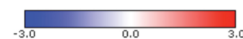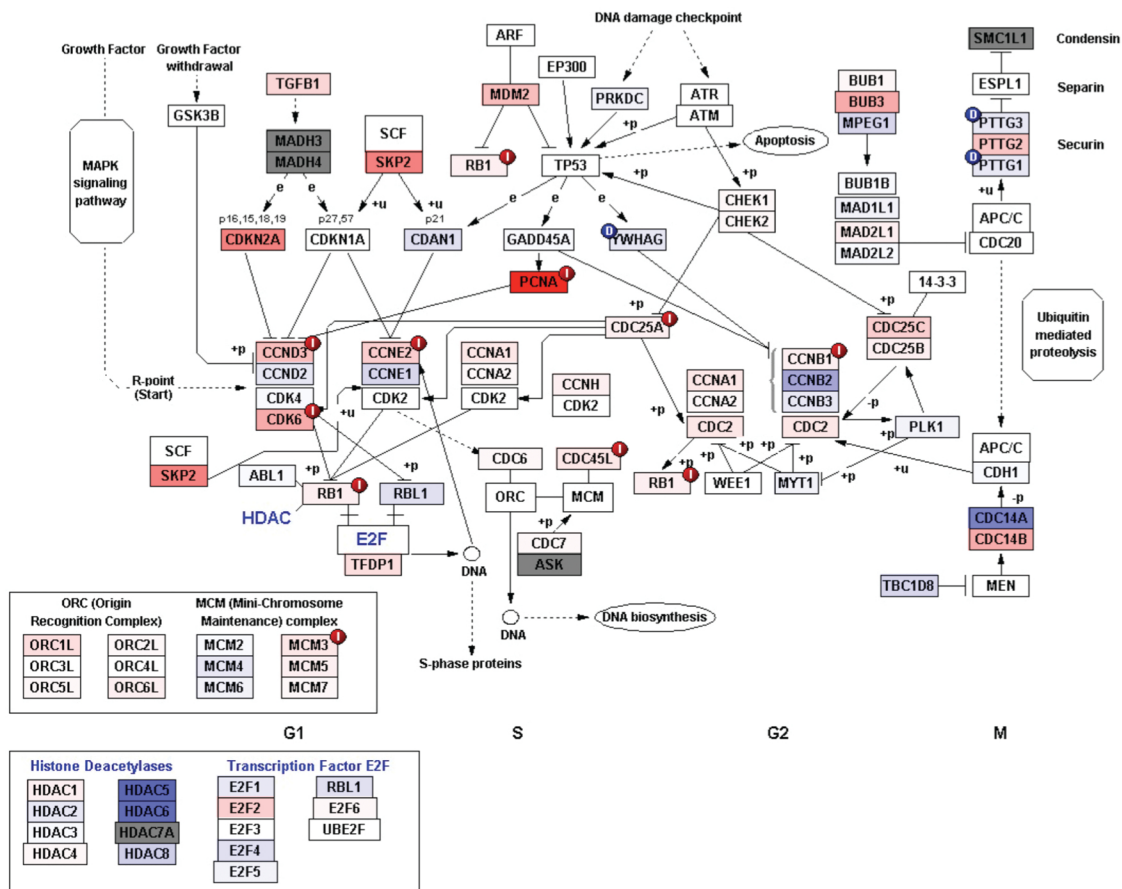

Supplement: Additional file 6: Figure S6 — Cell cycle genes affected in estrogen deprived cells. Red and blue boxes indicate up- and down regulated gene expression in response to estrogen deprivation. Small circles (red and blue) mark a statistically significant increase or decrease in expression. (A) MCF7 cells at 6 weeks after estrogen deprivation versus control cells (B) BT474 cells at 2 days after estrogen deprivation versus control cells. [file 1471-2407-13-473-S6.pdf]

A

**ESR1 probes**

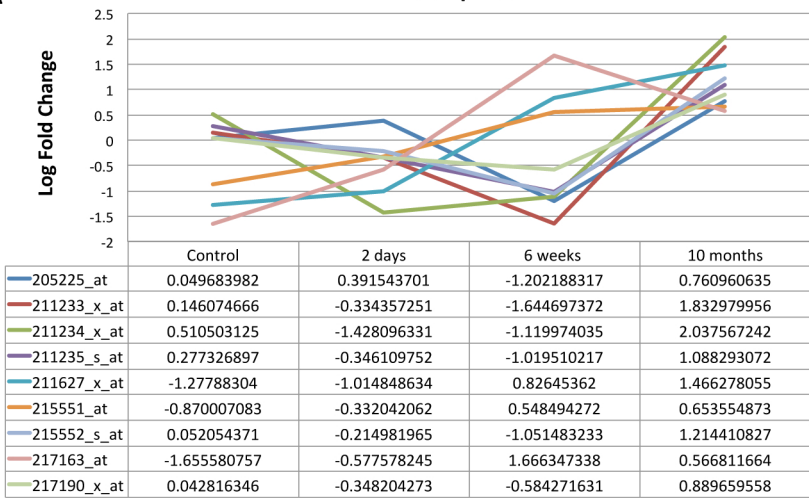

B

**MKI67 probes**

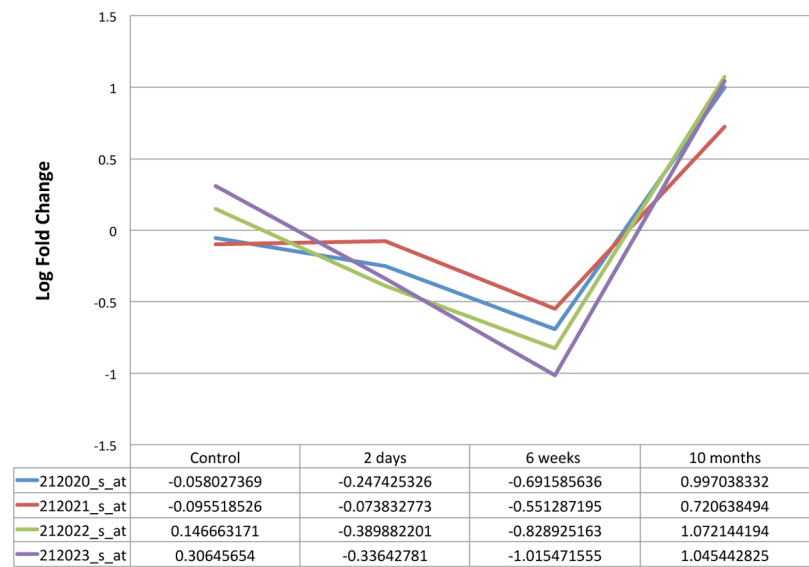

C

**ER genomic function genes**

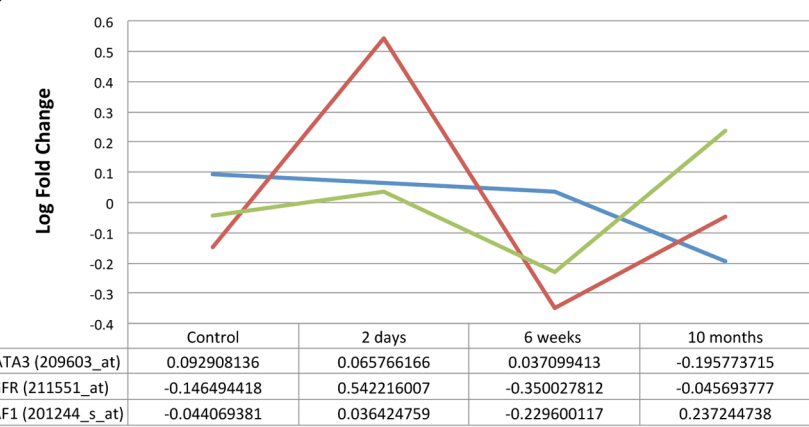

Supplement: Additional file 8: Figure S7 — The effect of LTED on selected probesets. Here, in order to put our results in context with other scientific publications we reproduced the probeset plots of Aguilar et al. (A) ESR1 affymetrix probesets (B) MKI67 affymetrix probesets (C) Genes related to ER genomic function. [file 1471-2407-13-473-S8.pdf]
