# Supplementary material for: Clinical instability of breast cancer markers is reflected in long-term in vitro estrogen deprivation studies
Source: BMC Cancer. 2013 Oct 11;13:473. doi: 10.1186/1471-2407-13-473 (PMC3852062; doi:10.1186/1471-2407-13-473)
Supplement: Additional file 7: Table S1 — Log fold change of cell cycle genes in MCF7 cells 2 days after estrogen deprivation versus control. This table displays all genes of the human KEGG annotated cell cycle pathway and their fold change after two days of estrogen deprivation relative to control, sorted according to p-value. Note, multiple affymetrix probes can map to the same gene. *Direction of change: I = Increase, D = Decrease and NC = No statistically significant change. [file 1471-2407-13-473-S7.pdf]

Affymetrix probes	Gene Symbol	MCF7 log fold change	Direction of change*	MCF7 p-value
224847_at	CDK6	1.3	I	0.00004
212672_at	ATM	-1	D	4E-05
203132_at	RB1	-0.5	D	4E-05
201456_s_at	BUB3	-0.4	D	4E-05
201746_at	TP53	-0.6	D	4E-05
203755_at	BUB1B	-0.8	D	4E-05
203418_at	CCNA2	-0.8	D	4E-05
213226_at	CCNA2	-0.7	D	4E-05
214710_s_at	CCNB1	-0.5	D	4E-05
228729_at	CCNB1	-0.9	D	4E-05
205034_at	CCNE2	-0.8	D	4E-05
203213_at	CDC2	-0.6	D	4E-05
205167_s_at	CDC25C	-1	D	4E-05
202246_s_at	CDK4	-0.5	D	4E-05
203362_s_at	MAD2L1	-0.7	D	4E-05
220651_s_at	MCM10	-0.6	D	4E-05
203625_x_at	SKP2	-1.1	D	4E-05
211814_s_at	CCNE2	-0.6	D	4.6E-05
210416_s_at	CHEK2	-0.8	D	4.6E-05
201202_at	PCNA	-0.4	D	4.6E-05
210559_s_at	CDC2	-0.7	D	4.6E-05
204510_at	CDC7	-0.8	D	4.6E-05
204225_at	HDAC4	-0.6	D	5.4E-05
203968_s_at	CDC6	-0.7	D	5.4E-05
202284_s_at	CDKN1A	1.1	I	0.000054
205288_at	CDC14A	-1.4	D	7E-05
202107_s_at	MCM2	-0.4	D	7E-05
210983_s_at	MCM7	-0.6	D	7E-05
216237_s_at	MCM5	-0.4	D	9.2E-05
204857_at	MAD1L1	-0.6	D	0.000104
204126_s_at	CDC45L	-1	D	0.000136
243000_at	CDK6	0.8	I	0.000176
210742_at	CDC14A	-1.5	D	0.000228
221586_s_at	E2F5	-0.4	D	0.000228
205394_at	CHEK1	-0.8	D	0.000228
203214_x_at	CDC2	-0.5	D	0.00026
201555_at	MCM3	-0.2	D	0.000378
202455_at	HDAC5	0.8	I	0.000378
218284_at	SMAD3	0.6	I	0.000428
224848_at	CDK6	0.9	I	0.000428
203967_at	CDC6	-1	D	0.000546
1556269_at	MYT1	2.2	I	0.000614
204093_at	CCNH	-0.5	D	0.000692
1554768_a_at	MAD2L1	-0.6	D	0.000692
201755_at	MCM5	-0.6	D	0.000876
208694_at	PRKDC	-0.3	D	0.000876
223570_at	MCM10	-1.2	D	0.001104
201930_at	MCM6	-0.4	D	0.001104
209903_s_at	ATR	-0.2	D	0.001548
202870_s_at	CDC20	-0.2	D	0.001548
211347_at	CDC14B	3.7	I	0.002672
205296_at	RBL1	-0.7	D	0.002672
205393_s_at	CHEK1	-0.6	D	0.002672
204817_at	ESPL1	-0.3	D	0.003664
1555772_a_at	CDC25A	-3.5	D	0.004064
222962_s_at	MCM10	-1	D	0.004064

235287_at	CDK6	0.8	NC	0.005506
203693_s_at	E2F3	-0.2	NC	0.005506
210996_s_at	YWHAE	-0.5	NC	0.00671
201853_s_at	CDC25B	-0.2	NC	0.00671
224851_at	CDK6	0.3	NC	0.008146
209642_at	BUB1	-0.4	NC	0.00985
213523_at	CCNE1	0.5	NC	0.015576
217717_s_at	YWHAB	-0.2	NC	0.015576
1553759_at	MCM9	0.1	NC	0.017022
203554_x_at	PTTG1	-0.2	NC	0.017022
208795_s_at	MCM7	-0.2	NC	0.018584
238075_at	CHEK1	-0.6	NC	0.02209
215711_s_at	WEE1	0.6	NC	0.02209
205398_s_at	SMAD3	0.4	NC	0.033462
242939_at	TFDP1	-0.3	NC	0.033462
203725_at	GADD45A	0.4	NC	0.036256
200951_s_at	CCND2	0.9	NC	0.036256
201457_x_at	BUB3	-0.1	NC	0.039248
238977_at	MCM6	-0.6	NC	0.04951
237891_at	MDM2	3.4	NC	0.04951
1555003_at	RBL1	-2.1	NC	0.057532
226818_at	MPEG1	1.6	NC	0.057532
211803_at	CDK2	0.7	NC	0.066608
215757_at	PRKDC	3.3	NC	0.066608
204695_at	CDC25A	-0.5	NC	0.07157
200640_at	YWHAZ	0.3	NC	0.07157
202123_s_at	ABL1	0.4	NC	0.082402
228813_at	HDAC4	-0.2	NC	0.088298
231198_at	CDK6	1.9	NC	0.094528
234605_at	CDC14B	1	NC	0.094528
210317_s_at	YWHAE	-0.4	NC	0.101106
203957_at	E2F6	-0.1	NC	0.108044
217400_at	PCNA	-0.6	NC	0.115352
211300_s_at	TP53	-0.6	NC	0.115352
211722_s_at	HDAC6	3	NC	0.115352
205386_s_at	MDM2	-0.5	NC	0.123044
1555186_at	CDKN1A	-1.8	NC	0.131132
212142_at	MCM4	-0.4	NC	0.157874
1555004_a_at	RBL1	-0.2	NC	0.167652
215509_s_at	BUB1	-1.4	NC	0.177876
244504_x_at	ARF1	2.5	NC	0.188558
242105_at	CCNE1	0.3	NC	0.188558
210441_at	CDC14A	-1	NC	0.188558
209902_at	ATR	-0.2	NC	0.199704
203085_s_at	TGFB1	1.3	NC	0.211326
216914_at	CDC25C	0.9	NC	0.223428
1562028_at	CCND3	2.6	NC	0.223428
1565702_at	SMAD4	2.7	NC	0.249104
231534_at	CDC2	-1.5	NC	0.262686
205899_at	CCNA1	0	NC	0.306464
219673_at	MCM9	-0.2	NC	0.322076
204947_at	E2F1	-1	NC	0.338196
201130_s_at	CDH1	-0.4	NC	0.354824
210858_x_at	ATM	-0.3	NC	0.371962
232175_at	ARF1	0.1	NC	0.371962
226841_at	MPEG1	0.6	NC	0.389602
222036_s_at	MCM4	0	NC	0.389602
223909_s_at	HDAC8	0.4	NC	0.407742

201458_s_at	BUB3	-0.1	NC	0.407742
200953_s_at	CCND2	0.4	NC	0.426376
200065_s_at	ARF1	0.2	NC	0.426376
38707_r_at	E2F4	0.1	NC	0.443286
2028_s_at	E2F1	-0.1	NC	0.454486
242325_at	YWHAH	-0.7	NC	0.465098
203692_s_at	E2F3	-0.3	NC	0.465098
200952_s_at	CCND2	2.6	NC	0.465098
202645_s_at	MEN1	0.2	NC	0.465098
202240_at	PLK1	-0.2	NC	0.465098
215822_x_at	MYT1	-0.7	NC	0.48517
223234_at	MAD2L2	-0.1	NC	0.48517
232764_at	CCNB2	1.6	NC	0.505702
202221_s_at	EP300	-0.4	NC	0.505702
217373_x_at	MDM2	-0.5	NC	0.505702
234740_at	CDC14B	0.5	NC	0.526682
231481_at	CCNB3	-0.6	NC	0.548096
209112_at	CDKN1B	-0.1	NC	0.569934
236559_at	YWHAH	-0.4	NC	0.569934
205385_at	MDM2	0	NC	0.569934
211832_s_at	MDM2	0.2	NC	0.592178
208511_at	PTTG3	0	NC	0.592178
210567_s_at	SKP2	-0.1	NC	0.614812
1554322_a_at	HDAC4	2.5	NC	0.614812
204696_s_at	CDC25A	0.3	NC	0.708884
211540_s_at	RB1	-0.8	NC	0.733186
201131_s_at	CDH1	0.2	NC	0.75776
203626_s_at	SKP2	0.2	NC	0.75776
216275_at	BUB1	0.5	NC	0.75776
1553387_at	ATM	3.4	NC	0.75776
202248_at	E2F4	-0.4	NC	0.807624
210440_s_at	CDC14A	-1.8	NC	0.807624
216326_s_at	HDAC3	-0.2	NC	0.807624
205396_at	SMAD3	-0.4	NC	0.807624
233288_at	ATR	0.2	NC	0.807624
1565651_at	ARF1	0.3	NC	0.832866
1560161_at	CCNB2	1	NC	0.832866
225787_at	UBE2F	-0.1	NC	0.832866
210341_at	MYT1	1.4	NC	0.858282
212533_at	WEE1	0.2	NC	0.858282
229827_at	BUB3	0	NC	0.858282
215508_at	BUB1	0.8	NC	0.909532
202527_s_at	SMAD4	-0.2	NC	0.909532
208442_s_at	ATM	-0.2	NC	0.935312
209644_x_at	CDKN2A	-0.2	NC	0.935312
214557_at	PTTG2	0.3	NC	0.935312
200638_s_at	YWHAZ	0.2	NC	0.935312
217010_s_at	CDC25C	0.8	NC	0.96116
216277_at	BUB1	-0.8	NC	0.96116
204147_s_at	TFDP1	0.5	NC	0.96116
235725_at	SMAD4	0.1	NC	1
204526_s_at	TBC1D8	0.3	NC	1
235582_at	E2F2	-1.9	NC	1
221592_at	TBC1D8	0.3	NC	1
211348_s_at	CDC14B	0.3	NC	1
207039_at	CDKN2A	-0.9	NC	1
1554631_at	ATM	0.1	NC	1
232768_at	CCNB2	-0.2	NC	1

210743_s_at	CDC14A	0.2	NC	1
211804_s_at	CDK2	0.3	NC	1
207143_at	CDK6	-0.2	NC	1
213579_s_at	EP300	0.1	NC	1
209945_s_at	GSK3B	0	NC	1
201209_at	HDAC1	0.2	NC	1
201833_at	HDAC2	0	NC	1
229408_at	HDAC5	0	NC	1
206846_s_at	HDAC6	-0.1	NC	1
223345_at	HDAC8	-0.1	NC	1
223908_at	HDAC8	0.1	NC	1
233560_x_at	MCM8	0.8	NC	1
205397_x_at	SMAD3	0.2	NC	1
1565703_at	SMAD4	0	NC	1
202526_at	SMAD4	0.2	NC	1
203084_at	TGFB1	-0.4	NC	1
208743_s_at	YWHAB	0	NC	1
200693_at	YWHAQ	0	NC	1
212426_s_at	YWHAQ	0.2	NC	1
213699_s_at	YWHAQ	0.2	NC	1
1570352_at	ATM	3.4	NC	1
211156_at	CDKN2A	-0.1	NC	1
242141_at	HDAC2	-0.1	NC	1
207042_at	E2F2	0.3	NC	1
216224_s_at	HDAC6	-0.4	NC	1
241017_at	TBC1D8	0	NC	1
1559307_s_at	RBL1	0.8	NC	1
208750_s_at	ARF1	0	NC	1
222985_at	YWHAG	0	NC	1
225791_at	UBE2F	0.6	NC	1
224320_s_at	MCM8	0.1	NC	1
200639_s_at	YWHAZ	0.1	NC	1
217718_s_at	YWHAB	0.1	NC	1
204252_at	CDK2	0.1	NC	1
225783_at	UBE2F	0	NC	1
201700_at	CCND3	0.2	NC	1
231948_s_at	UBE2F	0	NC	1
228361_at	E2F2	0	NC	1
209974_s_at	BUB3	0.1	NC	1
201020_at	YWHAH	-0.1	NC	1
200641_s_at	YWHAZ	0.1	NC	1
202705_at	CCNB2	0	NC	1
210543_s_at	PRKDC	0	NC	1
212141_at	MCM4	0	NC	1
222037_at	MCM4	0	NC	1
212330_at	TFDP1	0.1	NC	1
242538_at	TFDP1	0	NC	1
38158_at	ESPL1	0	NC	1
