# Supplementary material for: Clinical instability of breast cancer markers is reflected in long-term in vitro estrogen deprivation studies
Source: BMC Cancer. 2013 Oct 11;13:473. doi: 10.1186/1471-2407-13-473 (PMC3852062; doi:10.1186/1471-2407-13-473)
Supplement: Additional file 9: Table S2 — Log fold change of cell cycle genes in BT474 cells 6 weeks after estrogen deprivation versus control. This table displays all genes of the human KEGG annotated cell cycle pathway and their fold change after 6 weeks of estrogen deprivation relative to control, sorted according to p-value. Note, multiple affymetrix probes can map to the same gene. *Direction of change: I = Increase, D = Decrease and NC = No statistically significant change. [file 1471-2407-13-473-S9.pdf]

Affymetrix probes	Gene Symbol	BT474 log fold change	Direction of change	BT474 p-value
38158_at	ESPL1	-0.6	D	2E-06
224847_at	CDK6	1.5	I	0.00004
203755_at	BUB1B	-1.3	D	4E-05
203418_at	CCNA2	-1.8	D	4E-05
213226_at	CCNA2	-1.5	D	4E-05
214710_s_at	CCNB1	-1.6	D	4E-05
228729_at	CCNB1	-1.7	D	4E-05
205034_at	CCNE2	-0.4	D	4E-05
203213_at	CDC2	-0.8	D	4E-05
205167_s_at	CDC25C	-1.7	D	4E-05
202246_s_at	CDK4	-0.6	D	4E-05
203362_s_at	MAD2L1	-1.2	D	4E-05
220651_s_at	MCM10	-1.4	D	4E-05
203625_x_at	SKP2	-0.6	D	4E-05
210559_s_at	CDC2	-0.9	D	4E-05
204510_at	CDC7	-0.8	D	4E-05
203968_s_at	CDC6	-1.3	D	4E-05
202107_s_at	MCM2	-0.8	D	4E-05
210983_s_at	MCM7	-0.9	D	4E-05
216237_s_at	MCM5	-1.5	D	4E-05
205394_at	CHEK1	-1.4	D	4E-05
203214_x_at	CDC2	-1	D	4E-05
203967_at	CDC6	-1.7	D	4E-05
208694_at	PRKDC	-0.9	D	4E-05
201930_at	MCM6	-1.1	D	4E-05
205393_s_at	CHEK1	-1.7	D	4E-05
201853_s_at	CDC25B	-0.7	D	4E-05
209642_at	BUB1	-1.6	D	4E-05
203554_x_at	PTTG1	-1.5	D	4E-05
208795_s_at	MCM7	-0.8	D	4E-05
201457_x_at	BUB3	-0.7	D	4E-05
200640_at	YWHAZ	-0.3	D	4E-05
222036_s_at	MCM4	-1	D	4E-05
202240_at	PLK1	-1.8	D	4E-05
223234_at	MAD2L2	-0.6	D	4E-05
212141_at	MCM4	-1.1	D	4E-05
222037_at	MCM4	-1.1	D	4E-05
212330_at	TFDP1	-0.6	D	4E-05
242538_at	TFDP1	-0.6	D	4E-05
202870_s_at	CDC20	-2	D	4.6E-05
210567_s_at	SKP2	-1.1	D	4.6E-05
200638_s_at	YWHAZ	-0.5	D	4.6E-05
210543_s_at	PRKDC	-1.1	D	4.6E-05
228813_at	HDAC4	0.6	I	0.000046
221586_s_at	E2F5	-0.8	D	5.4E-05
1554768_a_at	MAD2L1	-1.5	D	5.4E-05
208511_at	PTTG3	-1	D	6E-05
224851_at	CDK6	1.1	I	0.00006
238075_at	CHEK1	-1	D	8E-05
201755_at	MCM5	-1.1	D	0.00012
202705_at	CCNB2	-1.3	D	0.00012
1565651_at	ARF1	0.9	I	0.000156
201746_at	TP53	-1.2	D	0.000156
201202_at	PCNA	-0.4	D	0.000156
201555_at	MCM3	-0.4	D	0.000178
204126_s_at	CDC45L	-1	D	0.000228

200641_s_at	YWHAZ	-0.6	D	0.000294
217717_s_at	YWHAB	-0.3	D	0.000334
209974_s_at	BUB3	-0.4	D	0.000334
201020_at	YWHAH	-0.7	D	0.000334
204817_at	ESPL1	-0.5	D	0.000428
213523_at	CCNE1	-0.7	D	0.000428
210416_s_at	CHEK2	-0.7	D	0.000546
228361_at	E2F2	-0.8	D	0.000546
235725_at	SMAD4	0.4	I	0.000546
218284_at	SMAD3	0.8	I	0.000692
222962_s_at	MCM10	-1.2	D	0.001104
204695_at	CDC25A	-1.2	D	0.001104
231948_s_at	UBE2F	-0.5	D	0.001236
224848_at	CDK6	0.8	I	0.001384
238977_at	MCM6	-0.7	D	0.001384
242939_at	TFDP1	-1	D	0.001932
223570_at	MCM10	-1.1	D	0.002154
212142_at	MCM4	-2.1	D	0.002154
209902_at	ATR	-0.4	D	0.002154
205288_at	CDC14A	1.1	I	0.002402
203725_at	GADD45A	0.3	I	0.002402
204526_s_at	TBC1D8	0.4	I	0.002402
202455_at	HDAC5	0.4	I	0.003302
2028_s_at	E2F1	-0.4	D	0.003752
202527_s_at	SMAD4	-0.4	D	0.0045
202645_s_at	MEN1	-0.3	D	0.00498
204225_at	HDAC4	0.2	NC	0.005506
215509_s_at	BUB1	-5	NC	0.005506
1556269_at	MYT1	0.5	NC	0.006082
201700_at	CCND3	-0.8	NC	0.007398
215711_s_at	WEE1	-0.3	NC	0.008146
202284_s_at	CDKN1A	0.3	NC	0.011866
202221_s_at	EP300	0.3	NC	0.011866
214557_at	PTTG2	-1.3	NC	0.013006
225783_at	UBE2F	-0.4	NC	0.01424
232175_at	ARF1	0.7	NC	0.015576
225787_at	UBE2F	-0.6	NC	0.015576
235287_at	CDK6	0.8	NC	0.017022
210317_s_at	YWHAE	-0.6	NC	0.024048
243000_at	CDK6	0.6	NC	0.033462
204252_at	CDK2	-0.4	NC	0.042448
235582_at	E2F2	0.9	NC	0.04951
234605_at	CDC14B	-0.6	NC	0.057532
1560161_at	CCNB2	-1.6	NC	0.066608
217718_s_at	YWHAB	-0.3	NC	0.066608
209903_s_at	ATR	-0.2	NC	0.07157
205396_at	SMAD3	-0.9	NC	0.088298
211814_s_at	CCNE2	-0.3	NC	0.094528
204147_s_at	TFDP1	-0.2	NC	0.094528
200953_s_at	CCND2	0.4	NC	0.108044
231534_at	CDC2	-2.4	NC	0.115352
201130_s_at	CDH1	-0.3	NC	0.131132
211300_s_at	TP53	-1.1	NC	0.139626
236559_at	YWHAH	-3.2	NC	0.139626
215508_at	BUB1	-1.2	NC	0.139626
211722_s_at	HDAC6	-1.3	NC	0.148536
200639_s_at	YWHAZ	-0.2	NC	0.157874
204696_s_at	CDC25A	0	NC	0.188558

224320_s_at	MCM8	-0.4	NC	0.188558
204947_at	E2F1	-0.5	NC	0.199704
211832_s_at	MDM2	-0.5	NC	0.211326
225791_at	UBE2F	-0.8	NC	0.236018
1555772_a_at	CDC25A	-0.3	NC	0.249104
222985_at	YWHAG	-0.3	NC	0.276772
1553387_at	ATM	1.3	NC	0.291364
211540_s_at	RB1	0.6	NC	0.306464
1553759_at	MCM9	0.1	NC	0.354824
219673_at	MCM9	0.2	NC	0.354824
1565702_at	SMAD4	0.3	NC	0.354824
210742_at	CDC14A	0.3	NC	0.371962
205386_s_at	MDM2	-1.2	NC	0.407742
208750_s_at	ARF1	-0.2	NC	0.407742
205385_at	MDM2	-0.4	NC	0.426376
1555004_a_at	RBL1	-0.5	NC	0.445498
215757_at	PRKDC	-0.5	NC	0.465098
226818_at	MPEG1	1.5	NC	0.48517
234740_at	CDC14B	-0.2	NC	0.48517
1559307_s_at	RBL1	-0.7	NC	0.505702
202248_at	E2F4	0.5	NC	0.526682
207042_at	E2F2	-2.2	NC	0.526682
216224_s_at	HDAC6	-0.3	NC	0.526682
241017_at	TBC1D8	-0.6	NC	0.526682
229827_at	BUB3	1.3	NC	0.548096
221592_at	TBC1D8	2.6	NC	0.569934
210341_at	MYT1	0.2	NC	0.592178
211347_at	CDC14B	-1	NC	0.592178
200952_s_at	CCND2	-2.4	NC	0.614812
242141_at	HDAC2	-0.4	NC	0.614812
231481_at	CCNB3	0.2	NC	0.661178
231198_at	CDK6	0.6	NC	0.684874
201456_s_at	BUB3	-0.2	NC	0.75776
211348_s_at	CDC14B	-0.4	NC	0.75776
205398_s_at	SMAD3	0.5	NC	0.78258
233288_at	ATR	-0.6	NC	0.807624
38707_r_at	E2F4	-0.2	NC	0.822456
205296_at	RBL1	-0.3	NC	0.832866
211803_at	CDK2	-0.4	NC	0.909532
216914_at	CDC25C	-0.3	NC	0.909532
215822_x_at	MYT1	-0.1	NC	0.909532
207039_at	CDKN2A	0.3	NC	0.909532
211156_at	CDKN2A	1.4	NC	0.935312
208442_s_at	ATM	0.3	NC	0.935312
1570352_at	ATM	-0.1	NC	0.96116
1555003_at	RBL1	1	NC	0.987048
212533_at	WEE1	-0.2	NC	0.987048
204093_at	CCNH	0	NC	0.987048
217400_at	PCNA	0.8	NC	0.987048
212672_at	ATM	-0.1	NC	1
203132_at	RB1	0.1	NC	1
204857_at	MAD1L1	0.1	NC	1
203693_s_at	E2F3	0	NC	1
210996_s_at	YWHAE	-0.2	NC	1
200951_s_at	CCND2	0.1	NC	1
237891_at	MDM2	-2	NC	1
202123_s_at	ABL1	0	NC	1
203957_at	E2F6	0	NC	1

1555186_at	CDKN1A	0.5	NC	1
244504_x_at	ARF1	0	NC	1
242105_at	CCNE1	-0.1	NC	1
210441_at	CDC14A	0.2	NC	1
203085_s_at	TGFB1	-0.4	NC	1
1562028_at	CCND3	-0.2	NC	1
205899_at	CCNA1	-0.4	NC	1
210858_x_at	ATM	0.1	NC	1
226841_at	MPEG1	0.3	NC	1
223909_s_at	HDAC8	0	NC	1
201458_s_at	BUB3	0	NC	1
200065_s_at	ARF1	-0.1	NC	1
242325_at	YWHAH	0.1	NC	1
203692_s_at	E2F3	-0.1	NC	1
232764_at	CCNB2	-0.1	NC	1
217373_x_at	MDM2	0	NC	1
209112_at	CDKN1B	0	NC	1
1554322_a_at	HDAC4	0.1	NC	1
201131_s_at	CDH1	0	NC	1
203626_s_at	SKP2	1.3	NC	1
216275_at	BUB1	-0.5	NC	1
210440_s_at	CDC14A	-2.4	NC	1
216326_s_at	HDAC3	0	NC	1
209644_x_at	CDKN2A	0	NC	1
217010_s_at	CDC25C	2.2	NC	1
216277_at	BUB1	2.6	NC	1
1554631_at	ATM	0.1	NC	1
232768_at	CCNB2	0.1	NC	1
210743_s_at	CDC14A	-0.1	NC	1
211804_s_at	CDK2	-0.2	NC	1
207143_at	CDK6	1.3	NC	1
213579_s_at	EP300	0.1	NC	1
209945_s_at	GSK3B	-0.1	NC	1
201209_at	HDAC1	0	NC	1
201833_at	HDAC2	0	NC	1
229408_at	HDAC5	0.1	NC	1
206846_s_at	HDAC6	-0.2	NC	1
223345_at	HDAC8	0.1	NC	1
223908_at	HDAC8	0	NC	1
233560_x_at	MCM8	-1.6	NC	1
205397_x_at	SMAD3	-0.3	NC	1
1565703_at	SMAD4	0	NC	1
202526_at	SMAD4	0.4	NC	1
203084_at	TGFB1	0.1	NC	1
208743_s_at	YWHAB	-0.1	NC	1
200693_at	YWHAQ	-0.2	NC	1
212426_s_at	YWHAQ	-0.1	NC	1
213699_s_at	YWHAQ	-0.2	NC	1
