# Supplementary material for: Clinical instability of breast cancer markers is reflected in long-term in vitro estrogen deprivation studies
Source: BMC Cancer. 2013 Oct 11;13:473. doi: 10.1186/1471-2407-13-473 (PMC3852062; doi:10.1186/1471-2407-13-473)
Supplement: Additional file 10: Table S3 — MCF7 cells 2 days after estrogen deprivation versus control cells. The table represents the number of genes matching the 10 most commonly occurring GO terms in the GO molecular function, biological processes, and cellular component classes. The 300 genes with highest SLR were selected. [file 1471-2407-13-473-S10.pdf]

ID	GO category	System	Gene Number	p-value
Up-regulated Processes				
GO:0005886	plasma membrane	cellular component	64	1.80×10^{-07}
GO:0032501	multicellular organismal process	biological process	66	2.44×10^{-07}
GO:0048856	anatomical structure development	biological process	48	3.75×10^{-07}
GO:0003779	actin binding	molecular function	16	5.07×10^{-07}
GO:0008092	cytoskeletal protein binding	molecular function	19	8.53×10^{-07}
GO:0044459	plasma membrane part	cellular component	44	8.63×10^{-07}
GO:0030054	cell junction	cellular component	19	9.61×10^{-07}
GO:0005575	cellular component	cellular component	192	9.91×10^{-07}
GO:0044464	cell part	cellular component	183	1.08×10^{-06}
GO:0005623	cell	cellular component	183	1.09×10^{-06}
Down-regulated Processes				
GO:0006563	L-serine metabolic process	biological process	5	1.32×10^{-08}
GO:0019752	carboxylic acid metabolic process	biological process	21	1.38×10^{-07}
GO:0006082	organic acid metabolic process	biological process	21	1.56×10^{-07}
GO:0006760	folic acid and derivative metabolic process	biological process	5	4.25×10^{-07}
GO:0006520	amino acid metabolic process	biological process	14	4.90×10^{-07}
GO:0008652	amino acid biosynthetic process	biological process	7	6.15×10^{-07}
GO:0009070	serine family amino acid biosynthetic process	biological process	4	2.13×10^{-06}
GO:0003674	molecular function	molecular function	180	2.30×10^{-06}
GO:0006519	cellular amino acid and derivative metabolic process	biological process	15	2.52×10^{-06}
GO:0006564	L-serine biosynthetic process	biological process	3	4.22×10^{-06}
