# Supplementary material for: Clinical instability of breast cancer markers is reflected in long-term in vitro estrogen deprivation studies
Source: BMC Cancer. 2013 Oct 11;13:473. doi: 10.1186/1471-2407-13-473 (PMC3852062; doi:10.1186/1471-2407-13-473)
Supplement: Additional file 11: Table S4 — BT474 cells 2 days after estrogen deprivation versus control cells. The table represents the number of genes matching the 10 most commonly occurring GO terms in the GO molecular function, biological processes, and cellular component classes. The 300 genes with highest SLR were selected. [file 1471-2407-13-473-S11.pdf]

ID	GO category	System	Gene Number	p-value
Up-regulated Processes				
GO:0005783	endoplasmic reticulum	cellular component	31	3.85×10^{-08}
GO:0005789	endoplasmic reticulum membrane	cellular component	21	8.41×10^{-07}
GO:0044432	endoplasmic reticulum part	cellular component	21	1.11×10^{-06}
GO:0042175	nuclear envelope-endoplasmic reticulum network	cellular component	21	1.28×10^{-06}
GO:0012505	endomembrane system	cellular component	33	2.16×10^{-06}
GO:0031224	intrinsic to membrane	cellular component	87	2.36×10^{-06}
GO:0044425	membrane part	cellular component	95	2.71×10^{-06}
GO:0016021	integral to membrane	cellular component	85	3.70×10^{-06}
GO:0016020	membrane	cellular component	107	8.41×10^{-06}
GO:0005575	cellular component	cellular component	199	1.10×10^{-05}
Down-regulated Processes				
GO:0051272	positive regulation of cell motility	biological process	8	1.56×10^{-07}
GO:0042981	regulation of apoptosis	biological process	21	3.81×10^{-07}
GO:0043067	regulation of programmed cell death	biological process	21	4.63×10^{-07}
GO:0030335	positive regulation of cell migration	biological process	7	1.11×10^{-06}
GO:0019318	hexose metabolic process	biological process	10	1.12×10^{-06}
GO:0042127	regulation of cell proliferation	biological process	20	1.25×10^{-06}
GO:0050793	regulation of developmental process	biological process	26	1.33×10^{-06}
GO:0048518	positive regulation of biological process	biological process	>1000	1.34×10^{-06}
GO:0005996	monosaccharide metabolic process	biological process	226	1.49×10^{-06}
GO:0048522	positive regulation of cellular process	biological process	>1000	1.53×10^{-06}
