# Supplementary material for: Clinical instability of breast cancer markers is reflected in long-term in vitro estrogen deprivation studies
Source: BMC Cancer. 2013 Oct 11;13:473. doi: 10.1186/1471-2407-13-473 (PMC3852062; doi:10.1186/1471-2407-13-473)
Supplement: Additional file 12: Table S5 — Genes in common amongst those significantly altered in all three analysed datasets: MCF7 LTED culture, ER-silenced MCF7 cells and breast cancer patients treated with aromatase inhibitors. We determined the genes most significantly altered in three datasets; our MCF7 LTED samples (control vs. 6 weeks, pvalue cutoff = 0.004), a publically available dataset of MCF7 cells where the ER has been silenced (control vs. silenced, pvalue cutoff = 0.004, GSE27473) and a publically available dataset of breast cancer patients treated with aromatase inhibitors (GSE5462). We then determined the genes in common amongst those significantly altered in all three studies and present them here divided into those up and down regulated. [file 1471-2407-13-473-S12.pdf]

Gene Symbol	Gene Description	Chromosome	GenBank	Gene ID
Up-regulated Genes				
HTRA1	HtrA serine peptidase 1	10	NM_002775	5654
SNAI2	snail homolog 2 (Drosophila)	8	AI572079	6591
TGFBR2	transforming growth factor, beta receptor II (70/80kDa)	3	D50683	7048
WWTR1	WW domain containing transcription regulator 1	3	BF674349	25937
Down-regulated Genes				
RACGAP1	Rac GTPase activating protein 1	12	AU153848	29127
TPBG	trophoblast glycoprotein	6	NM_006670	7162
FKBP4	FK506 binding protein 4, 59kDa	12	NM_002014	2288
SERPINA3	serpin peptidase inhibitor	14	NM_001085	12
TFF3	trefoil factor 3 (intestinal)	21	NM_003226	7033
BAZ1A	bromodomain adjacent to zinc finger domain, 1A	14	AA102574	11177
NRIP1	nuclear receptor interacting protein 1	21	AI824012	8204
CA12	carbonic anhydrase XII	15	BF752277	771
PAICS	phosphoribosylaminoimidazole carboxylase	4	AA902652	10606
IRS1	insulin receptor substrate 1	2	NM_005544	3667
ABAT	4-aminobutyrate aminotransferase	16	AF237813	18
